# A Novel Highly Reproducible and Lethal Nonhuman Primate Model for Orthopox Virus Infection

**DOI:** 10.1371/journal.pone.0010412

**Published:** 2010-04-29

**Authors:** Marit Kramski, Kerstin Mätz-Rensing, Christiane Stahl-Hennig, Franz-Josef Kaup, Andreas Nitsche, Georg Pauli, Heinz Ellerbrok

**Affiliations:** 1 Robert Koch-Institut, Berlin, Germany; 2 German Primate Center (DPZ), Göttingen, Germany; University of California San Francisco, United States of America

## Abstract

The intentional re-introduction of Variola virus (VARV), the agent of smallpox, into the human population is of great concern due its bio-terroristic potential. Moreover, zoonotic infections with Cowpox (CPXV) and Monkeypox virus (MPXV) cause severe diseases in humans. Smallpox vaccines presently available can have severe adverse effects that are no longer acceptable. The efficacy and safety of new vaccines and antiviral drugs for use in humans can only be demonstrated in animal models. The existing nonhuman primate models, using VARV and MPXV, need very high viral doses that have to be applied intravenously or intratracheally to induce a lethal infection in macaques. To overcome these drawbacks, the infectivity and pathogenicity of a particular CPXV was evaluated in the common marmoset (*Callithrix jacchus*).

A CPXV named **calpox virus** was isolated from a lethal orthopox virus (OPV) outbreak in New World monkeys. We demonstrated that marmosets infected with calpox virus, not only via the intravenous but also the intranasal route, reproducibly develop symptoms resembling smallpox in humans. Infected animals died within 1–3 days after onset of symptoms, even when very low infectious viral doses of 5×10^2^ pfu were applied intranasally. Infectious virus was demonstrated in blood, saliva and all organs analyzed.

We present the first characterization of a new OPV infection model inducing a disease in common marmosets comparable to smallpox in humans. Intranasal virus inoculation mimicking the natural route of smallpox infection led to reproducible infection. *In vivo* titration resulted in an MID_50_ (minimal monkey infectious dose 50%) of 8.3×10^2^ pfu of calpox virus which is approximately 10,000-fold lower than MPXV and VARV doses applied in the macaque models. Therefore, the calpox virus/marmoset model is a suitable nonhuman primate model for the validation of vaccines and antiviral drugs. Furthermore, this model can help study mechanisms of OPV pathogenesis.

## Introduction

After the worldwide vaccination program conducted by the World Health Organization (WHO), smallpox was eradicated in 1980 [1], and soon after eradication general vaccination with Vaccinia virus (VACV) against smallpox was discontinued. The threat of intentional re-introduction of Variola virus (VARV), the etiological agent of smallpox, into the human population by terrorists remains of concern today because of the easy transmission of the virus from person to person, a high mortality rate after infection with the virus and the low or non-existent smallpox immunity in the majority of the human population. Over the last years an increasing number of humans infected with other orthopox viruses (OPV) such as Cowpox (CPXV) and Monkeypox virus (MPXV) was observed, indicating the zoonotic potential of OPV species [2]–[5]. Outbreaks of MPXV have repeatedly been reported from Central and Western Africa. In 2003, an outbreak of human MPXV infection occurred in the mid-western United States due to the inadvertent importation of MPXV by a shipment of rodents from West Africa [3], [6]. Although several squirrel species are suspected to be natural reservoirs, the animal reservoir for MPXV in Africa remains unknown [7]. In case MPXV were to establish a reservoir status in a susceptible North American rodent species such as prairie dogs [6], [8], the consequences for public health would be considerable.

CPXV seems to be limited to Europe and Central Asia with a wide host range including humans [9],[10]. CPXV is endemic in wild rodents like bank voles (*Clethrionomys glareolus*) and wood mice (*Apodemus sylvaticus*). However, CPXV is also sporadically detected in rats (*Rattus norvegicus*) [11], [12]. Domestic cats can become infected by virus transmission from CPXV infected rodents and are therefore commonly recognized as host for CPXV and are responsible for the majority of human CPXV infections [13], [14]. In addition, CPXV has been isolated from a variety of zoo animals [15]–[17]. Increasing travel activity and importation of exotic animal species increase the risk of poxvirus transmissions outside their usual geographical distribution. Therefore diagnostics might often be delayed which has important implications.

The majority of these infections, particularly CPXV infections, are mild and generally self-limiting with low morbidity. But immunocompromised individuals may show a variety of complications including generalised infection and death [18], [19]. On the other hand, MPXV shows a significant morbidity and childhood mortality in Africa [3].

The risk of a bioterrorist attack with VARV led to a vaccination campaign in the USA in 2002. Persons with a potential risk of OPV (VARV) exposure like military personnel and health-care workers were vaccinated with the vaccine strain Dryvax. But as a high rate of adverse effects associated with smallpox vaccination has been observed, hardly any other country has recommended a general vaccination against smallpox [20], [21].

Consequently, research and development of new vaccines and therapeutic agents for the prevention and treatment of poxvirus infections is called for. During the period between vaccination and antibody development protection against an OPV infection is not ensured, neither does post-exposure vaccination necessarily provide protection against a lethal infection [22], [23]. Antiviral therapies can fill this gap and thereby complement vaccination by reducing viral load regardless of the immune status. Today, no FDA-approved antiviral drugs for the treatment of orthopox virus infections are available [23], [24]. But some antiviral compounds like Cidofovir and ST-246 showed an OPV-suppressing effect in cell culture and, as therapeutic drug, in a variety of animal models [25]–[27]. ST-246 was recently used very efficiently in the treatment of a 2-year-old child with a severe eczema vaccinatum [28].

To license drugs or vaccines for prevention or therapy of human infectious diseases like smallpox, these compounds must show adequate protection in the case of a challenge with the pathogen in one or more animal models. Based on this legislation, the United States Food and Drug Administration (FDA) recommend the testing of drugs or vaccines directed against such human diseases in at least two animals, one using a non-rodent species. This should ideally be a nonhuman primate. Although *in vivo* efficacy and safety studies of vaccines and antiviral drugs should be done in humans, this is not possible in the case of VARV/MPXV due to ethical reasons. Therefore, nonhuman primates are used for the testing of vaccines and antiviral compounds as these animals are closely related to humans [29]. Currently the most common animal models for studying the pathogenesis of OPV are the Ectromelia virus (ECTV)/mouse model and the VACV/mouse model. Infection with VARV and MPXV induces clinical disease in two macaque species, i.e. cynomolgus (*Macaca fascicularis*) and rhesus macaques (*Macaca mulatta*). However, these nonhuman primate models suffer from major limitations: only high infectious doses applied via an atypical route of infection reproducibly induce a severe or lethal course of infection. In cynomolgus macaques at least 10^8^ plaque forming units (pfu) of VARV have to be injected intravenously (i.v.) to induce a smallpox-like disease [30], and rhesus macaques have to be inoculated intravenously with at least 2×10^7^ pfu of MPXV to evoke symptoms [31], [32]. For the cynomolgus macaque/MPXV model different inoculation routes like intravenous (i.v.), intranasal (i.n.), subcutaneous (s.c.) and intratracheal (i.t.) application are described. But in this model also high viral doses of 10^6^–10^7^ pfu are necessary for the induction of smallpox-like symptoms, whereas animals that were given a dose of <10^6^ pfu (i.n.) often survived depending on the MPXV strain [23], [32], [33]. Although the clinical picture in this model is similar to naturally acquired smallpox in humans where patients survived depending on the virus strain, using non-lethal infection models would not be sufficient to demonstrate the efficacy of newly developed vaccines and therapeutic agents. Hence, a nonhuman primate model with a lethal outcome would be advantageous. Additionally, working with MPXV and VARV is methodologically challenging, as bio-safety level (BSL) three or four conditions are required. Moreover, macaques are relatively big animals and the costs of maintenance are high compared to other animal models for OPV infections. Therefore, the development of a safe and reproducible OPV/nonhuman primate model which allows the induction of a disease comparable to human smallpox is highly recommended.

Thirty Callithrix as well as Tamarin monkey species died within a week after onset of clinical symptoms in a colony of 80 New World monkeys in a private zoo in Lower Saxony, Germany [34]. Histological and electron microscopical investigations revealed the presence of OPV particles in different organs of deceased animals. PCR and sequence analysis of selected genes confirmed these findings and allowed a tentative grouping of this isolate to the OPV species CPXV [34]. Samples from 6 of these animals were available for laboratory confirmation. Virus isolation was performed from skin tissue of deceased animals on Vero E6 and Hep2 cells. The isolate was tentatively named calpox virus according to its host *Callithrix jacchus*.

Here we describe the establishment of a new nonhuman primate model for the study of OPV infections using the calpox virus, a new CPXV, and the common marmoset *Callithrix jacchus* which overcomes major limitations of existing primate models.

## Results

### Survival for different routes of infection

In order to determine whether the virus isolated was capable to induce the disease observed during the natural outbreak, five animals (group I) were inoculated intravenously (i.v.) with a viral dose of 1.25×10^7^ pfu (animal I-a and I-b) or with 1.0×10^7^ pfu (animal I-c, I-d and I-e). All animals died between day four and day seven post infection (p.i.) (table 1). The symptoms observed resembled those documented in the outbreak. As the i.v. virus application resulted in a very rapid disease progression, only blood samples at the time of death could be analyzed. High viral loads with titers between 10^6^ and 10^9^ genome equivalents (GE)/mL blood were measured (table 1).

**Table 1:** Survival, calpox viral load in blood and tissues and seroconversion after intravenous and intranasal inoculation of different doses of calpox virus.

animals		virus inoculation		detection of symptoms	survival [days p.i.]	viral load in blood [[calpox GE per1 ml blood]/[calpox mRNA copies per 10^6^ c-myc mRNA copies] a			IgM^b^	viral load in tissue^c^	
group	no.	route	dose [pfu]	day p.i.		day detectable p.i.	initial viral load	final viral load		tissues with ≤10^5^	tissues with ≥10^7^
**intravenous inoculation**											
I	a	i.v.	1.25×10^7^		4	na	na	4.2×10^8^/3.5×10^7^	—		sk, he, lu, li, sp, ln
	b				6	na	na	3.3×10^9^/1.4×10^8^	—		sk, he, lu, li, ki, sp, ln, bl, si, es
	c	i.v.	1.0×10^4^		5	na	na	1.0×10^6^/5.1×10^6^	—	bl, st, es, si, co, br	sk, lu, li, sp
	d				7	na	na	8.0×10^8^/4.9×10^7^	—		sk, lu, li, sp, ln
	e				7	na	na	6.9×10^8^/2.5×10^7^	—	si	sk, lu, li, sp, ln, es
**intranasal inoculation**											
II	a	i.n.	2.3×10^6^		10	10	nd/nd	4.0×10^6^/3.8×10^7^	—	bl, st, si, co, br	sk, sp, es, to
	b				9	7	4.0×10^5^/4.5×10^7^	1.4×10^8^/3.8×10^7^	—	si, co, es, br	sk, lu, sp, ln, to
III	a	i.n.	3.5×10^5^		9	7	1.7×10^5^/1.4×10^7^	9.7×10^7^/1.2×10^8^	—	si, co, br	sk, lu, sp, tr
	b				9	7	8.9×10^4^/6.9×10^6^	3.6×10^7^/3.1×10^8^	—	bl, si, co, es	sk, sp, to
**intranasal calpox virus titration**											
IV	a	i.n.	3.5×10^4^	14	14	7	9.5×10^3^/3.2×10^4^	1.3×10^9^/3.5×10^7^	—	he, bl, si	sk, lu, ki, sp, ln
	b			11	14	7	1.5×10^4^/2.2×10^5^	2.2×10^9^/2.1×10^8^	—	si, br	sk, he, lu, li, ki, sp, ln, tr
V	a	i.n.	3.5×10^3^	11	14	7	1.1×10^4^/5.6×10^4^	2.8×10^7^/1.9×10^8^	+	es, co, br	sk, lu, sp, ln
	b			14	14	10	nd/nd	3.5×10^7^/1.1×10^8^	+	he, bl, st, si, co, br	sk, ln
VI	a	i.n.	5.0×10^2^	14	14	7	2.7×10^2^/7.3×10^2^	1.3×10^8^/7.3×10^7^	+	bl, br	sk, lu, li, sp, ln, tr
	b			none	42^d^	nd	nd/nd	nd/nd	—	na	na
VII	a	i.n.	1.0×10^2^	none	42^d^	nd	nd/nd	nd/nd	—	na	na
	b			none	42^d^	nd	nd/nd	nd/nd	—	na	na
**A re-inoculation of animals VI b, VII a and b** e											
VI	b	i.n.	5.0×10^2^	none	28^d^	nd	nd/nd	nd/nd	—	na	na
VII	a			14	14	7	1.9×10^5^/1.7×10^5^	3.6×10^7^/1.1×10^7^	—		sk, lu, li, sp, ln, es
VII	b			none	28^d^	nd	nd/nd	nd/nd	—	na	na
**B 2^nd^ re-inoculation of animals VI b and VII b** e											
VI	b	i.n.	3.5×10^3^	10	12	7	4.4×10^4^/4.2×10^6^	2.1×10^9^/2.8×10^6^	—	st, si	sk, lu, li, sp, ln
VII	b			none	28^df^	nd	nd/nd	nd/nd	—	all tissues tested were negative	

a calculation of the copy numbers for both, calpox virus and c-myc is based on the mean value of duplicate measurements and a respective quantified plasmid standard for each real-time PCR assay.

b seroconversion, detection of IgM antibodies in plasma by immunofluorescence assay at the time point of death or at pre-determined end of experiment (surviving animals).

c viral load was expressed as calpox GE/10^6^ c-myc genomic DNA; all tissues that were analyzed are indicated, from infected animals all tissues tested were positive. Viral load was defined as low = equal or below 10^5^GE, or high = equal or above 10^7^GE. Tissue abbreviations: **sk**: skin; **he**: heart; **lu**: lung; **li**: liver, **sp**: spleen, **ki**: kidney; **ln**: lymph node (axillaris, mesenterialis, submandibularis); **bl**: bladder; **st**: stomach; **es**: esophagus; **si**: small intestine; **co**: colon; **br**: brain; **to**: tonsils; **tr**: trachea.

d pre-determined endpoint of experiment for animals without sign of infection.

e animals not infected during previous experiment(s) were re-infected.

f animal was sacrificed at end of experiment.

**i.v.**: intravenous, **i.n.**: intranasal; **na**: no sample available; **nd**: not detectable; the detection limit for both assays was 10 copies; **GE**: genome equivalents (genomic DNA).

After reproduction of the symptoms in marmosets by i.v. inoculation of calpox virus, two other infection routes, i.e. the oropharyngeal and intranasal (i.n.) inoculation were investigated. An oropharyngeal inoculation was performed by applying approximately 10^7^ pfu of the virus next to the tonsillar region. As only one out of five animals showed symptoms and died (data not shown) it was decided to refrain from a more detailed investigation of this infection route. For intranasal inoculation two different virus preparations were investigated: a virus stock prepared after three passages in Hep2 cells (virus stock A) and the original cell culture isolate (virus stock B). Two marmosets were inoculated with 2.3×10^6^ pfu (group II) (2×50 µl, titer of stock A 2.3×10^7^ pfu/mL) and two marmosets with 3.5×10^5^ pfu (group III) (2×50 µl, titer of stock B: 3.5×10^6^ pfu/mL).

**Figure 1:**
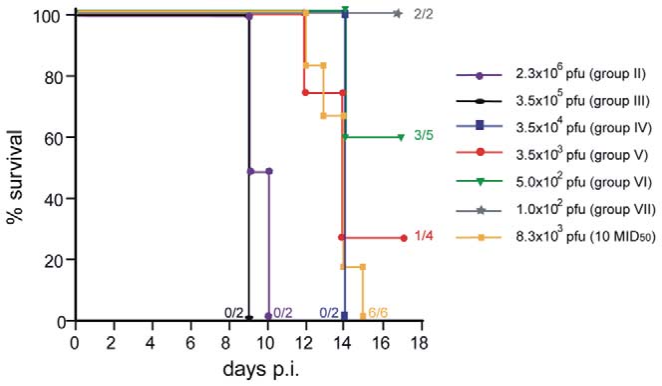
Kaplan-Meier plot showing the effect of different doses of calpox virus inoculated intranasally (i.n.) on the survival of marmosets. Survival post infection (p.i.) is expressed as percentages. Ratios given on the right side indicate numbers of surviving animals versus numbers of animals inoculated. Doses as low as 8.3×10^3^ pfu led to 100% mortality.

All four marmosets died between day 9 and 10 p.i. (figure 1). Blood samples were taken on day seven p.i. and at the time of death. On day seven p.i. calpox virus was detected by real-time PCR in three out of four animals (II-b, III-a, III-b) with viral loads around 10^5^ calpox virus GE/mL blood. At that time none of the animals showed clinical signs of a disease. All four animals had a final load of ≥10^6^ calpox virus GE/mL blood at the time of death (table 1). No difference in the course of infection was observed for either virus preparation using the i.n. infection route.

### Determination of calpox virus minimal lethal dose

To determine the MID_50_ (minimal monkey infectious dose where 50% of the animals die) for the i.n. infection route, four groups (group IV–VII) of two marmosets each were inoculated i.n. with 3.5×10^4^, 3.5×10^3^, 5×10^2^ and 1×10^2^ pfu of calpox virus, respectively.

In animals of group IV (3.5×10^4^ pfu), group V (3.5×10^3^ pfu) and in one animal of group VI (5×10^2^ pfu) the titer of calpox virus in blood continuously increased from day seven p.i. onwards. All five animals died on day 14 p.i. with high viral loads in blood (table 1 and figure 1).

The second animal of group VI (5×10^2^ pfu) as well as both animals of group VII (1×10^2^ pfu) did not become infected, as defined by lack of clinical symptoms as well as viremia and seroconversion within the observation period of 42 days. All three surviving marmosets (animals VI-b, VII-a and-b) were re-inoculated intranasally with 5×10^2^ pfu of calpox virus, resulting in infection of one animal (VII-a) (table 1, re-inoculation). The other two animals (VI-b and VII-b) did not show signs or markers of infection during the pre-determined study time frame of 28 days and were re-inoculated with calpox virus with a higher dose of 3.5×10^3^ pfu. One of the two animals (VI-b) became infected and died on day 12 p.i. with approximately 10^9^ calpox GE/mL blood. Animal VII-b survived without developing any clinical symptoms, and neither calpox virus nor antibodies against OPV were detected in blood or tissue at necropsy (table 1, 2^nd^ re-inoculation).

Using the VACMAN 3.1 program [35], the MID_50_ of calpox virus was calculated. This program is generally used for statistical analysis of primate trials working with very small animal numbers as well as virus dilutions other than 1∶10 for virus titration. Standard statistical analysis programs do not take into account the effect of all the experimental imponderabilities inherent in this model, like virus dilution, virus stability or genetic variation between animals. At a dose of 5×10^2^ pfu corresponding to a virus stock dilution of 1∶700 two out of five animals died, reaching the *in vivo* endpoint of calpox virus via the i.n. route. From this data an MID_50_ of 8.3×10^2^ pfu of calpox virus was estimated.

### Reproducibility of infection with 10 MID_50_

The number of animals used for *in vivo* titration was too small to statistically support the data. Therefore, we infected six marmosets i.n. with a lethal dose of ten MID_50_, corresponding to 8.3×10^3^ pfu. All six animals died between day 12 and 15 p.i. (figure 1). Calpox virus DNA was detectable in the blood of all marmosets as early as day seven p.i., and the copy numbers rapidly increased towards death to final values of 10^9^ calpox GE/mL blood (data not shown).

### Clinical observations

Intravenous virus inoculation induced clinical symptoms like breathing difficulties, languishment and anorexia in five of five animals (100%) only one day before death. Macroscopic inspection of all deceased animals revealed few small papular skin lesions with a diameter of 2–3 mm on face, breast and inner sides of the thighs, which were often accompanied by focal hemorrhages. After i.n. inoculation the first clinical signs of infection (breathing difficulties, nasal discharge, languishment and anorexia) were similar to i.v.-infected animals and also appeared only one to two days before death. Characteristic of all i.n.-infected animals were edema with focal and petechial bleeding in the larynx and/or thorax area and multiple skin lesions scattered on face, abdomen and thighs as well as edema in the larynx. Lymph nodes draining the areas of the skin lesions were marked by severe lymphatic hyperplasia (further description of the pathology will be publish elsewhere). Quality and timing of clinical symptoms were independent of the viral dose and appeared one to two days before death. There was no recovery from the disease after virus was detected in blood (regarded as successful infection).

### Determination of calpox virus load in different tissues

**Figure 2:**
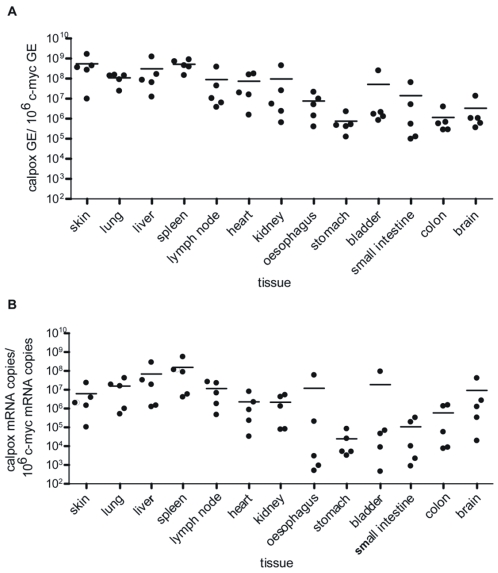
Distribution of calpox virus in different tissues. Virus was determined in different tissues of infected animals and is shown exemplarily for i.v. infected animals of group I determined by real-time PCR. **A**) calpox virus genome equivalents (GE) (viral DNA copy number)/10^6^ c-myc GE (DNA copy number of cellular gene marker); **B**) calpox virus mRNA copies/10^6^ c-myc mRNA copies; calculation of the copy numbers for calpox virus as well as c-myc is based on the mean value of duplicate measurements and a respective plasmid standard for each real-time PCR assay; lines indicate the median value for all 5 i.v.-infected marmosets (group I); GE: genome equivalents.

High levels of calpox virus DNA were detectable by quantitative real-time PCR in all tissues collected from all deceased marmosets independent of the route of infection or viral dose (figure 2 A shows an example for i.v. inoculation, table 1). The highest viral loads were measured in skin, spleen, lung, lymph node, tonsils and trachea (between 10^7^ and 10^8^ calpox virus GE/10^6^ c-myc GE). Lower viral loads were generally found in bladder, stomach, small intestine, esophagus, colon and brain (10^4^–10^5^ calpox virus GE/10^6^ c-myc GE) (table 1). In agreement with the data from viral DNA quantification, the highest RNA expression levels of calpox virus with up to 10^7^ calpox virus mRNA copies/10^6^ c-myc mRNA copies were found in lung, spleen, lymph node and skin (exemplarily shown for group I (i.v.) in figure 2 B), and the lowest calpox virus mRNA copy numbers were found in stomach, small intestine, esophagus and colon (∼10^4^ calpox virus mRNA copies/10^6^ c-myc mRNA copies). For both inoculation routes the highest viral loads were observed in lymphoid organs, indicating a progressive infection and replication of the virus in these organs, whereas calpox virus replicated less efficiently in organs of the digestion tract and the brain.

**Figure 3:**
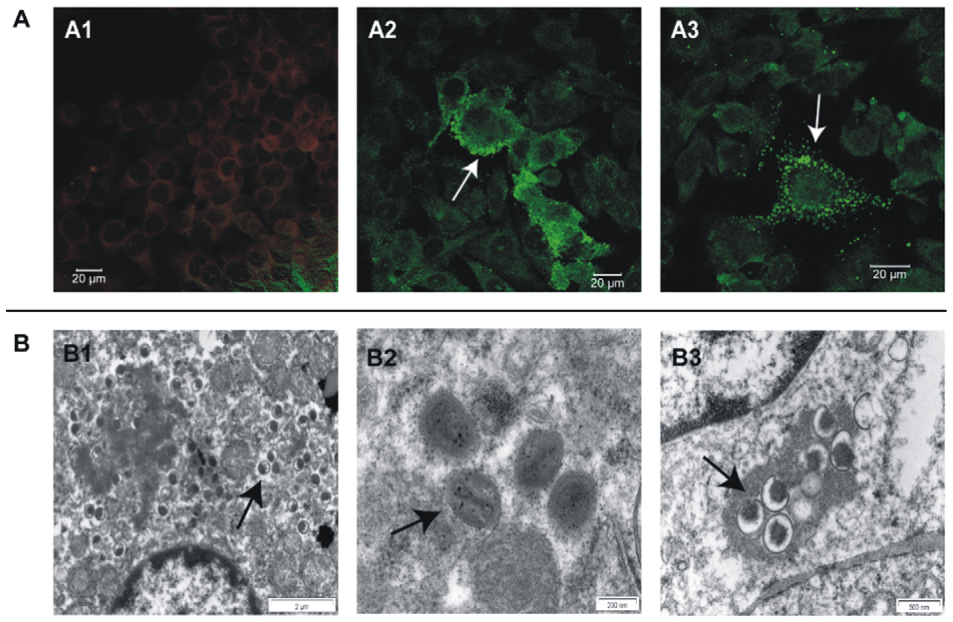
Analysis of antibodies and virus particles in infected animals. **Section A:** Determination of orthopox virus-specific IgM antibodies. IgM antibodies were determined in the serum of infected marmosets at the time of death (day 14 p.i.), calpox virus-infected Hep2 cells were incubated with serum (1∶10 diluted) and visualized with anti-human IgM-FITC labeled antibody, 1∶63 magnification; **A1**) negative serum; **A2**) animal V-a; **A3**) animal VI-a, bars correspond to 20 µm; **Section B:** Electron micrographs of mature and immature calpox virus particles in different tissues of animal V-b; **B1**) immature virus particles in liver tissue; **B2**) mature virus particles in lung tissue and **B3**) immature virus particles in spleen tissue; arrows indicate virus particles.

**Figure 4:**
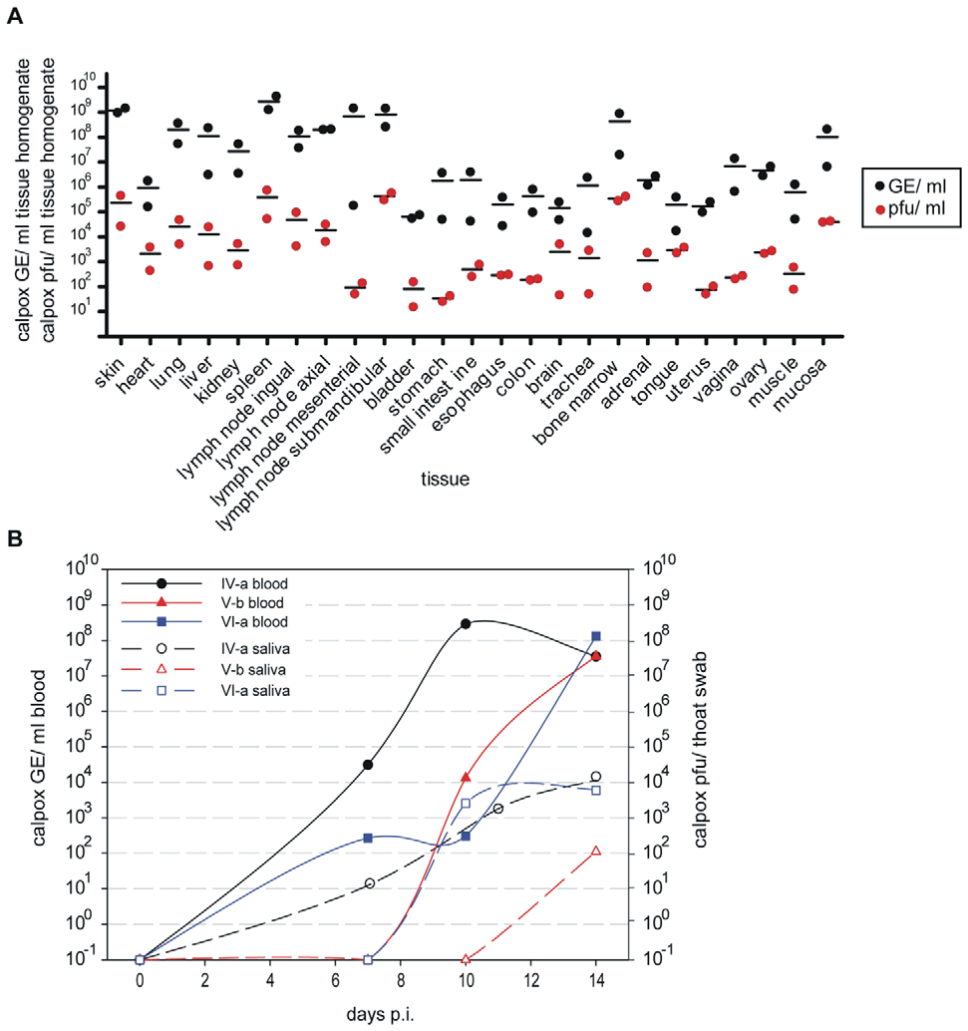
Detection of calpox virus in tissues and saliva. **A**) comparison of infectious calpox virus titer and calpox virus genome equivalents (GE) per ml tissue homogenate for two animals infected with 3.5×10^3^ (V-a) and 5.0×10^2^ pfu (VI-a) (line indicates the median value for animals V-a and VI-a); for real-time PCR: calculation of the copy numbers for calpox virus is based on the mean value of duplicate measurements and a respective plasmid standard; **B**) infectious calpox virus in saliva (normalization approximated by using throat swab as the denominator) compared to GE in blood (during course of infection) for three different infectious doses (group IV: 3.5×10^4^, V: 3.5×10^3^ and VI: 5.0×10^2^ pfu); pfu: plaque forming unit; p.i.: post infection; GE: genome equivalents.

Electron microscopy revealed typical brick-shaped mature and immature OPV virus particles in various tissues (figure 3, section B) also confirming successful infection. Infectious virus was quantified in tissues, using the plaque assay, and compared to the results obtained by quantitative PCR using the same tissue preparation and normalized to 1 mL tissue homogenate. Infectious calpox virus was detected in all tissues. The infectious virus titer of a tissue was about three to five logs lower than the titer of GE for all inoculation doses (figure 4 A, results of animal V-a infected with 3.5×10^3^ and VI-a 5×10^2^ pfu are shown as examples).

### Serology

Plasma samples of all virus-inoculated animals were screened for IgM and IgG antibodies, using an immunofluorescence assay, as well as for neutralizing antibodies, using a plaque reduction neutralization test (PRNT). After i.v. or i.n. infection with high doses of virus, no virus-specific antibodies could be found. This was expected as a surviving time of approximately 10 days p.i. is too short for developing OPV-specific antibodies.

Three out of eight marmosets inoculated with lower doses of calpox virus (animals V-a, V-b and VI-a) seroconverted with maximal IgM antibody titers of 1∶80 that were only detectable at the time of death at 14 days p.i. (figure 3, section A). Neither IgG nor neutralizing antibodies were detectable in any of the animals (data not shown). Supposedly, all infected animals died before the development of a specific IgG antibody response.

### Viral load in saliva

To investigate whether virus transmission could occur via droplets from aerosolized saliva, throat swabs and blood samples were collected simultaneously and analyzed for infectious calpox virus. The appearance of infectious calpox virus in saliva was delayed by approximately three days when compared to that in blood and was first detectable on day 10 (figure 4 B) in the majority of the animals which became infected and had survived up to this point in time. The amount of infectious virus per throat swab was difficult to normalize because the volume of saliva collected could not be quantified (saliva in throat swab was resolved in 300 µl D-MEM medium). In addition, the saliva was not cell-free and in consequence cell-associated virus might have been co-detected. However, infectious calpox virus titers increased parallel to viral loads in blood, reaching the highest levels at the time of death (figure 4 B). These results underline a potential virus transmission from animal to animal via virus-containing droplets as it has been described in naturally occurring smallpox infection in man.

## Discussion

As already shown for other nonhuman primate models [30], [31], [36], the i.v. inoculation route is a non-physiological route of infection which bypasses the mucosa and circumvents a primary replication of the virus in regional lymph nodes and the successive systemic spread via the lymphatic system, altogether representing the first viremia. Since the natural disease progression is altered in i.v. inoculation models [29], it is unclear how antiviral efficiency tested in i.v. models correlates with antiviral efficacy in naturally occurring human diseases. As human VARV infection normally occurs via airborne transmission, the i.n. calpox virus application was established in common marmosets mimicking this major natural route.

Here we demonstrated that the calpox virus isolate could produce a lethal disease in common marmosets which in a variety of features resembles smallpox rather than CPXV infection in humans, i.e., an airborne generalized lethal infection with lesions distributed all over the body compared to a localized self-limting infection usually occurring through skin lesions. Furthermore, in contrast to other OPV/nonhuman primate models, a low dose (MID_50_ of 8.3×10^2^ pfu) of cell-free virus applied i.n. reproducibly infected the animals even only statistically insufficient animal numbers were used for the determination of the MID_50_. Ten MID_50_ corresponding to 8.3×10^3^ pfu led to a lethal infection in all animals. The onset of clinical symptoms depended on the viral dose whereas the symptoms themselves were dose independent and generally appeared very shortly before death. There was no recovery from the infection in any of the animals when virus was detected in blood as well as after onset of symptoms. Furthermore, all animals lacking any clinical signs also failed to show markers of infection like antibodies against OPV antigens or detection of viral genomes by PCR. It seems like the immune response in marmosets is altered. Seroconversion is delayed and was only seen in three out of eight marmosets which survived up to 14 days p.i. This is in contrast to naturally occurring non-hemorrhagic smallpox infections in humans where IgG antibodies titers were detectable about 18 days p.i., respectively [37]. Moreover, after i.v. or i.n. infection with high doses of calpox virus, no virus-specific antibodies could be found. This is explained by the short surviving time of approximately 10 days p.i. which is probably too short for developing OPV-specific antibodies. Similar observations like delayed, reduced or even failing antibody response are reported for hemorrhagic-type smallpox patients [38]. In humans generally a sub-lethal infection results in the production of a protective immune response. This was not seen for the calpox virus/marmosets model. Due to the lack of statistical support of the data the alteration of the marmoset immune response after calpox virus infections needs further investigation.

Infectious calpox virus was found in the saliva of all diseased marmosets, with kinetics similar to those in blood. Therefore, the intranasal inoculation caused a clinical outcome probably capable of spreading the virus via a natural route of transmission among other animals before clinical symptoms became apparent – the same route of virus transmission that applies to human smallpox infection.

Independent of infection route and inoculated viral dose, high copy numbers of virus genomic DNA and virus-specific mRNA indicating active replication were found in a broad spectrum of organs and tissues, showing the wide cell tropism of calpox virus. The general involvement of multiple organs is similar to lethal human MPXV and VARV infections where virus is found in various organs [38]. The massive virus replication across all organs was probably associated with organ dysfunction and multisystem failure already shown for VARV infections in cynomolgus macaques [30].

In the calpox virus/marmoset model the time period between onset of clinical signs and death of the marmosets appears to be foreshortened compared to human smallpox. In contrast to severely diseased humans infected with VARV or MPXV [4], [38], [39]–[41], only a limited number of small skin lesions were observed and sparingly scattered over the bodies of the test animals, independent of the infection route. In other nonhuman primate models the number of skin lesions is part of the clinical score and points towards the disease outcome. This is different in the marmoset model although all infected marmosets developed skin lesions. However, all infected animals developed a clinically apparent infection which always resulted in the death of the animals. In no case was recovery from the disease observed. This is in contrast to the natural infection with VARV and MPXV, where only a fraction of the patients with clinical signs of infection had a lethal outcome [41]. Animals surviving inoculation of very low doses of calpox virus showed neither immunological nor virological markers of an infection, suggesting no take of the virus. A clinical score for the signs of illness defining the severity of the infection might be helpful and important, but does not seem to be necessary in the marmoset model in which any clinical symptom ends up in a lethal outcome. The readout for success or failure of any prophylactic or therapeutic interventions is simply the death rate in the respective study arm after viral exposure, making assessments of any measure much easier compared to other nonhuman primate models or in other animal models.

A weakness of other OPV nonhuman primate models is that the virus has to be administered in very high doses to induce fatal disease [23], [33], [42]–[44]. In contrast, the MID_50_ of calpox virus is approximately 10,000 times lower than that determined in other nonhuman primate model for OPV infections. An exception is the cynomolgus monkey/MPXV model where inhalation of 10,000 to 148,000 pfu of aerosolized MPXV in a modified Henderson head only exposure chamber induced lethal infection. However, technical and safety measures for this approach are considerable. Moreover, a successful infection with calpox virus is reproducibly achieved when 10 MID_50_ (8.3×10^3^ pfu of calpox virus) are administered intranasally. The low MID_50_ and the i.n. inoculation route used in the calpox virus/marmoset model mimic naturally acquired VARV and MPXV infections in humans.

Furthermore, epidemiologic investigations of the natural calpox virus outbreak in the colony of New World monkeys [34] gave no evidence of transmission of the virus to humans (Broll et al., unpublished). Experimental work with calpox virus, tentatively grouped to the OPV species CPXV, can be done under BSL-2 conditions. Advantages of the marmoset model over other nonhuman primate models include the small size of the animals, reasonably sized housing space and conditions, easy handling, a high reproduction rate in captivity, their inexpensive keeping, their economic purchase price and the fact that no wild populations are endangered [44]. Moreover, the common marmoset has already been used as a model for a wide spectrum of experimental research on human diseases [45]–[51].

Summarized, the calpox virus/marmoset model is an important addition and alternative to the primate models for OPV infection currently used because it overcomes the limitations of these models like the requirement of high viral doses applied by an unnatural route. Therefore, it is a valuable and suitable tool to evaluate the efficacy of new vaccination strategies and antiviral therapies for the prevention or treatment of human OPV infections.

## Materials and Methods

### Ethical statement

Marmosets (Callithrix jacchus) were bred and housed at the German Primate Centre under standard conditions according to the German Animal Welfare Act which complies with the European Union Directive 86/609/ECC and the European Union guidelines on the welfare of non-human primates used in research.

The study was performed in accordance with the regulations of the German Animal Protection Law. This includes statement of the responsible animal welfare officer of the German Primate Centre and authorization by the regional governmental veterinary authorities. The authorization process implies the hearing of the application and ethical judgement by an independent animal protection board which is composed of six persons from animal protection organisations and scientific community. The experiments were supervised by local governmental veterinary authorities. The approvals by the governmental veterinary authorities of Lower Saxony, Germany, have the reference numbers: 33.11.425-04-019/07; 33.425027/08-07.05; 33.42502/08-02.04.

All animals were inspected twice per day by the veterinarians of the German Primate Centre. In cases of suffering animals were treated with analgetic drugs or otherwise the experiment was stopped by humane killing with subsequent post mortem analysis.

### Animals, virus inoculation and sampling

Two to six years old adults of either sex were used. Housing conditions were 25°C, 60% humidity and single cages (130 cm×53 cm×80 cm) for each animal, with acoustical contact to each other. For virus application marmosets were anesthetized by injecting 0.1 mL of a mixture of 1 mg xylazine (Rompun®, Bayer AG, Leverkusen, Germany), 5 mg atropine (Boehringer, Ingelheim, Germany), 5 mg ketamine (Ketalar®, Parke-Davis, USA) per 200g body weight into the hamstring muscle. Calpox virus was administered i.v. in a maximal volume of 500 µl into the *vena saphena*. Oropharyngeal virus application was performed by dropping 100 µl virus-containing saline close to the tonsillar region. Intranasal infection was carried out as described elsewhere [52], [53] by pipetting 50 µl virus-containing saline into each nostril. Blood was collected under ketamine anesthesia by puncture of the femoral vein at regular intervals. Faucal swabs were obtained by swabbing the deeper pharyngeal area with a cotton-wool swab saturated with phosphate buffered saline (PBS). Thereafter, the swab was squeezed out in 300 µl DMEM medium and the fluid stored at −80°C until further use.

### Virus and cells

Calpox virus was propagated in Hep2 cells (ECACC: 86030501) in Dulbecco's modified Eagle's medium (DMEM) (Gibco) with 5% fetal calf serum and 1% glutamine at 37°C in a 5% CO_2_ humidified atmosphere. Infected cells were incubated for approximately four days until a pronounced cytopathic effect was observed. Supernatant and cells were harvested from infected cultures. After freeze-thawing and intensive vortexing with glass beads for better cell disruption the suspension was centrifuged for 10 min at 200 g to pellet the cell debris. The virus-containing supernatant was overlaid onto a 30% sucrose cushion and ultracentrifuged for 3 h at 3.4×10^3^ g. The pelleted virus was re-suspended in a small volume of 0.9% NaCl (approximately 0.5 mL/tube). The infectivity titer of the calpox virus stock was determined by a plaque assay on Vero E6 (ECACC 85020205) cells grown in DMEM supplemented with 10% fetal calf serum and 1% glutamine. Virus titers were expressed as plaque forming units (pfu) per mL.

### Isolation of calpox virus DNA and RNA from whole blood

50 or 100 µl EDTA blood was used for DNA extraction using the QIAamp viral Blood Kit (Qiagen, Hilden, Germany) according to the manufacturer's protocol. DNA was eluted in the same volume as the initial blood volume. For the isolation of RNA from blood a preceding erythrocyte lysis step was performed with a red cell lysis buffer (0.144 M NH_4_Cl, 1 mM NaHCO_3_ in H_2_O). RNA from blood cells was isolated using the RNeasy Mini Kit (Qiagen, Hilden, Germany) following the manufacturer's instructions. To avoid DNA contamination in RNA samples, a DNA digestion was additionally performed for 40 min at 37°C using the Ambion TURBO DNase Kit according to the manufacturer's protocol (Ambion, Applied Biosystems Business, Darmstadt, Germany).

### Isolation of calpox virus DNA and RNA from tissues

Tissue samples of a defined weight were homogenized in PBS using the FastPrep machine (MP Biomedicals, Eschwege, Germany). Aliquots of 100 µl homogenates were used for RNA and DNA extraction and for plaque assay. For the preparation of total DNA from tissue the QIAamp viral Blood Kit and for total RNA preparation the RNeasy Mini Kit was used. Similar to RNA extraction from blood cells a DNA digestion was performed for 40 min at 37°C using the Ambion TURBO DNase Kit according to the manufacturer's protocol (Ambion, Applied Biosystems Business, Darmstadt, Germany) to avoid DNA contamination in RNA samples. RNA was tested for DNA contamination by performing cDNA syntheses without reverse transcriptase followed by a c-myc real-time PCR.

cDNA was generated by reverse transcription (RT) in a total volume of 20 µl containing 10 µl RNA, 500 ng calf thymus DNA (Sigma Aldrich, Hamburg, Germany), 500 ng oligo dT_(18)_ primer (Metabion, Martinsried, Germany) which was incubated at 65°C for 5 min before adding 4 µl 5× buffer, 2 µl 0.1 M DTT, 0.4 µl 25 mM dNTP and 200 U Superscript II (Invitrogen, Karlsruhe, Germany). Afterwards samples were incubated at 37°C for 50 min and at 70°C for 10 min. For each RNA sample a negative RT reaction was performed in which the reverse transcriptase Superscript II was replaced by water to prove that the obtained RNA was DNA free.

### Determination of viral load by real-time PCR

For virus detection and quantification a calpox virus-specific real-time PCR assay (forward:5′-gTCTTTCTCgTTTACCAAgTgC; reverse:5′- ACAgAgAAAACATTTAAggATgAATCTATA; mgb-probe:Fam-TAgCTCCgTTTATTTTgTTA-NQ-mgb) was established. It showed no cross reactivity to other orthopox viruses, which might be important for later vaccination experiments. A c-myc-specific real-time PCR (forward:5′-gCCAgAggAggAACgAgCT; reverse:5′-ggg CCTTTTCATTgTTTTCCA; probe: 5′-FAM-TgCCCTgCgTgACCAgATCC-TAMRA) was used for relative quantification to standardize the number of cells in the samples. For absolute real-time PCR quantification the amplicons of the target regions for calpox virus and c-myc were cloned into a TOPO-TA vector (Invitrogen, Karlsruhe, Germany). Plasmids were quantified in a photospectrometer and 10-fold serial dilutions were used for calibration curves. All samples were measured in duplicate for both assays. The mean value of both C_T_ values was used for calculation of the copy numbers. Viral DNA quantities were expressed as calpox virus genome equivalents (GE) per mL blood or per 10^6^ copies of the c-myc gene for tissue samples. The calpox virus mRNA copy numbers were calculated per 10^6^ c-myc copies, too. For all real-time PCR assays the PlatinumTaq polymerase (Invitrogen, Karlsruhe, Germany) was used.

### Plaque assay

Infectious calpox virus in saliva and tissue homogenates was determined by plaque assay and expressed as pfu per mL. Briefly, tenfold serial dilutions of the samples (100 µl) were incubated with 100 µl of a Vero E6 cell suspension for 4 hours at 37°C and 5% CO_2_ in 24-well plates. After incubation cells were overlaid with 200 µl 1.6% carboxymethylcellulose (CMC) and incubated for another four days. Cell supernatant was aspirated and cells were fixed in 1 mL 3.7% formaldehyde for 30 min, stained with naphthalene blue black (1 g naphthol blue black [Sigma-Aldrich, Deisenhofen, Germany], 13.6 g sodium acetate, 60 mL glacial acetic acid [both Merck, Darmstadt, Germany], ad 1 l ddH_2_O) and washed once with water. The virus titer (pfu/mL) was calculated based on plaque numbers counted for each dilution step.

### Plaque reduction neutralization test (PRNT)

Plasma and serum samples were incubated for 30–60 min at 56°C to inactivate virus and/or complement. Twofold serial dilutions of the plasma samples were mixed with an equal volume of calpox virus. After an incubation for 1 h at 37°C and 5% CO_2_, 100 µl serum–virus mixture was added to pre-seeded Vero E6 cells in 100 µl cell culture medium. Virus was allowed to adsorb for 1 h at 37°C and 5% CO_2_. Cells were overlaid with 200 µl of 1.6% CMC and incubated for further four days at 37°C and 5% CO_2_. Medium was aspirated and fixation and staining of cells were done identically to the plaque assay. The number of plaques/well was counted and the titer for 50% plaque reduction was calculated in comparison to the virus titer obtained for negative marmoset serum which served as control.

### Immunofluorescence assay

To detect virus-specific antibodies in the plasma of infected marmosets, plasma samples were heat treated for 30–60 min at 56°C to inactivate virus as well as complement components. Subsequently, slides coated with calpox virus-infected Hep2 cells (fixed with acetone) were incubated for 1 h at 37°C with twofold serial dilutions of plasma. For IgM determination IgGs were removed using Mastsorb absorb (Mast Diagnostica, Reinfeld, Germany) before the plasma was incubated with the antigen. After two washing steps with 1× PBS a goat anti-human IgG FITC-labeled antibody (for IgG determination) or goat anti-human IgM FITC-labeled antibody (for IgM determination) (both Caltag, Burlingame, CA, USA) was used as second antibody. Evans Blue (Sigma-Aldrich, Deisenhofen, Germany) was added to stain the cytoplasm of the cells. Slides were analyzed by fluorescence microscopy.
